# Bis{2-eth­oxy-6-[2-(ethyl­ammonio)ethyl­imino­meth­yl]phenolato}thio­cyanato­zinc(II) nitrate

**DOI:** 10.1107/S1600536808029590

**Published:** 2008-09-20

**Authors:** Hui-Rui Guo

**Affiliations:** a College of Chemistry and Biological Engineering, Yichun University, Yichun Jiangxi 336000, People’s Republic of China

## Abstract

The title complex, [Zn(NCS)(C_13_H_20_N_2_O_2_)_2_]NO_3_, consists of a complex mononuclear Zn^II^ cation and a nitrate counter-anion. The Zn^II^ ion is five-coordinate in a trigonal-bipyramidal geometry. The thio­cyanate N atom and two O atoms from two Schiff bases define the equatorial plane, and the two imine N atoms from the same two Schiff bases occupy the axial positions, with an N—Zn—N angle of 175.98 (7)°. The amine N atoms of the Schiff base ligands are protonated and are not involved in the coordination to the metal. The dihedral angle between the two substituted benzene rings is 87.7 (2)°. The nitrate counter-ions are linked to the cations through N—H⋯O hydrogen bonds.

## Related literature

For background on Schiff base complexes, see: Samanta *et al.* (2007 ▶); Ghosh *et al.* (2006 ▶); Correia *et al.* (2006 ▶); Cai *et al.* (2006 ▶); Zhang *et al.* (2007 ▶). For Zn^II^ complexes with biological properties, see: Berg & Shi (1996 ▶); Tarafder *et al.* (2002 ▶); Osowole *et al.* (2008 ▶); Chohan & Kausar (1993 ▶). For related structures, see: Eltayeb *et al.* (2008 ▶); Odoko *et al.* (2006 ▶); Tatar *et al.* (2002 ▶).
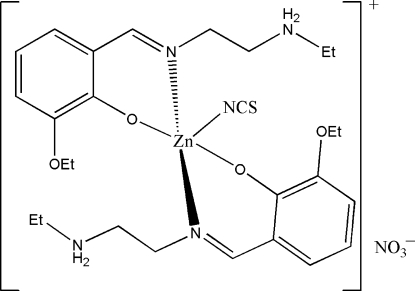


## Experimental

### 

#### Crystal data

[Zn(NCS)(C_13_H_20_N_2_O_2_)_2_]NO_3_
*M* *_r_* = 658.08
Monoclinic, 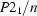

*a* = 12.619 (3) Å
*b* = 15.596 (3) Å
*c* = 16.373 (4) Å
β = 105.942 (3)°
*V* = 3098.5 (12) Å^3^
*Z* = 4
Mo *K*α radiation
μ = 0.91 mm^−1^
*T* = 298 (2) K
0.32 × 0.30 × 0.28 mm

#### Data collection

Bruker SMART CCD area-detector diffractometer
Absorption correction: multi-scan (*SADABS*; Sheldrick, 1996 ▶) *T*_min_ = 0.759, *T*_max_ = 0.784
25309 measured reflections
6704 independent reflections
4943 reflections with *I* > 2σ(*I*)
*R*_int_ = 0.039

#### Refinement

*R*[*F* ^2^ > 2σ(*F* ^2^)] = 0.040
*wR*(*F* ^2^) = 0.102
*S* = 1.03
6704 reflections
383 parameters
H-atom parameters constrained
Δρ_max_ = 0.37 e Å^−3^
Δρ_min_ = −0.24 e Å^−3^

### 

Data collection: *SMART* (Bruker, 1998 ▶); cell refinement: *SAINT* (Bruker, 1998 ▶); data reduction: *SAINT*; program(s) used to solve structure: *SHELXS97* (Sheldrick, 2008 ▶); program(s) used to refine structure: *SHELXL97* (Sheldrick, 2008 ▶); molecular graphics: *SHELXTL* (Sheldrick, 2008 ▶); software used to prepare material for publication: *SHELXTL*.

## Supplementary Material

Crystal structure: contains datablocks global, I. DOI: 10.1107/S1600536808029590/bh2194sup1.cif

Structure factors: contains datablocks I. DOI: 10.1107/S1600536808029590/bh2194Isup2.hkl

Additional supplementary materials:  crystallographic information; 3D view; checkCIF report

## Figures and Tables

**Table 1:** Hydrogen-bond geometry (Å, °)

*D*—H⋯*A*	*D*—H	H⋯*A*	*D*⋯*A*	*D*—H⋯*A*
N2—H2*B*⋯O5	0.90	1.96	2.843 (3)	166
N2—H2*A*⋯O3	0.90	1.98	2.772 (2)	146
N2—H2*A*⋯O4	0.90	2.37	3.022 (3)	129
N4—H4*A*⋯O7^i^	0.90	2.22	3.050 (3)	153
N4—H4*A*⋯O5^i^	0.90	2.50	3.080 (3)	122
N4—H4*B*⋯O1	0.90	1.71	2.606 (2)	171
N4—H4*B*⋯O2	0.90	2.55	3.051 (3)	116

Symmetry code: (i) 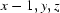.

**Fig. 1.**
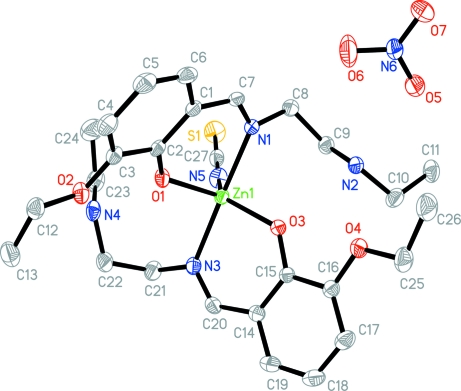
The structure of the title complex, showing 30% probability displacement ellipsoids and the atomic numbering scheme. H atoms have been omitted for clarity.

**Fig. 2.**
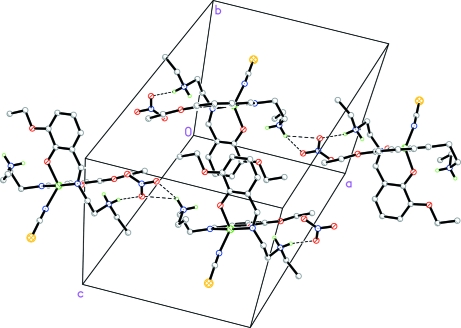
A part of the packing structure of the title complex.

## supplementary crystallographic information

### Comment

The synthesis and structural investigation of Schiff base complexes have attracted much attention due to their interesting structures and wide potential applications (Samanta *et al.*, 2007; Ghosh *et al.*, 2006; Correia *et al.*, 2006; Cai *et al.*, 2006; Zhang *et al.*, 2007). Zn^II^ complexes play an important role in biological systems (Berg & Shi, 1996; Tarafder *et al.*, 2002; Osowole *et al.*, 2008; Chohan & Kausar, 1993). In this paper, the crystal structure of a new Zn^II^ complex, (I), with the Schiff base 2-ethoxy-6-[(2-ethylammonioethylimino)methyl]phenol and thiocyanate as ligands is reported.

The asymmetric unit of (I) (Fig. 1) consists of a mononuclear Zn^II^ complex cation and a nitrate counteranion. The Zn^II^ ion is five-coordinate in a trigonal-bipyramidal geometry, with one thiocyanate N atom and two O atoms from two Schiff bases defining the base-plane, and with two imine N atoms from two Schiff bases occupying the axial positions. The coordination bond lengths and angles (Table 1) are comparable with those found in related structures (Eltayeb *et al.*, 2008; Odoko *et al.*, 2006; Tatar *et al.*, 2002). The dihedral angle between the two substituted benzene rings is 87.7 (2)°. The nitrate counterions are linked to the complex cations through intermolecular N—H···O hydrogen bonds (Table 2, Fig. 2).

### Experimental

The Schiff base was synthesized by condensing *N*-ethylethane-1,2-diamine with 3-ethoxysalicylaldehyde. The compound (0.236 g, 1.0 mmol), ammonium thiocyanate (0.076 g, 1.0 mmol), and Zn(NO_3_)_2_.6H_2_O (0.297 g, 1.0 mmol) were heated in ethanol (30 ml) for two hours. The resulting yellow precipitate was washed with cold ethanol and dried in air. Yellow single crystals of the complex suitable for X-ray diffraction were obtained by recrystallization in methanol at room temperature over a week.

### Refinement

All H-atoms were placed in calculated positions (C—H 0.93 to 0.97 Å, N—H 0.90 Å) and were included in the refinement in the riding model approximation, with *U*_iso_(H) set to 1.2 to 1.5*U*_eq_(C) and 1.2*U*_eq_(N). A rotating group model was used for the methyl groups.

### Crystal data

**Table:**

[Zn(NCS)(C_13_H_20_N_2_O_2_)_2_]NO_3_	*F*(000) = 1384
*M**_r_* = 658.08	*D*_x_ = 1.411 Mg m^−^^3^
Monoclinic, *P*2_1_/*n*	Mo *K*α radiation, λ = 0.71073 Å
Hall symbol: -P 2yn	Cell parameters from 5860 reflections
*a* = 12.619 (3) Å	θ = 2.4–25.0°
*b* = 15.596 (3) Å	µ = 0.91 mm^−^^1^
*c* = 16.373 (4) Å	*T* = 298 K
β = 105.942 (3)°	Block, yellow
*V* = 3098.5 (12) Å^3^	0.32 × 0.30 × 0.28 mm
*Z* = 4	

### Data collection

**Table:**

Bruker SMART CCD area-detector diffractometer	6704 independent reflections
Radiation source: fine-focus sealed tube	4943 reflections with *I* > 2σ(*I*)
graphite	*R*_int_ = 0.039
ω scans	θ_max_ = 27.0°, θ_min_ = 1.8°
Absorption correction: multi-scan (SADABS; Sheldrick, 1996)	*h* = −15→15
*T*_min_ = 0.759, *T*_max_ = 0.784	*k* = −19→19
25309 measured reflections	*l* = −20→20

### Refinement

**Table:**

Refinement on *F*^2^	Primary atom site location: structure-invariant direct methods
Least-squares matrix: full	Secondary atom site location: difference Fourier map
*R*[*F*^2^ > 2σ(*F*^2^)] = 0.040	Hydrogen site location: inferred from neighbouring sites
*wR*(*F*^2^) = 0.102	H-atom parameters constrained
*S* = 1.03	*w* = 1/[σ^2^(*F*_o_^2^) + (0.0499*P*)^2^ + 0.4058*P*] where *P* = (*F*_o_^2^ + 2*F*_c_^2^)/3
6704 reflections	(Δ/σ)_max_ < 0.001
383 parameters	Δρ_max_ = 0.37 e Å^−^^3^
0 restraints	Δρ_min_ = −0.24 e Å^−^^3^

### Fractional atomic coordinates and isotropic or equivalent isotropic displacement parameters (Å^2^)

**Table:**

	*x*	*y*	*z*	*U*_iso_*/*U*_eq_	
Zn1	0.16270 (2)	0.137705 (16)	0.239060 (17)	0.03736 (10)	
S1	0.23354 (8)	0.13520 (6)	0.53956 (5)	0.0790 (3)	
O1	0.06063 (12)	0.04409 (9)	0.19068 (10)	0.0431 (4)	
O2	−0.10767 (13)	−0.02591 (11)	0.08270 (10)	0.0489 (4)	
O3	0.24094 (12)	0.19615 (10)	0.16454 (10)	0.0422 (4)	
O4	0.36595 (13)	0.23273 (11)	0.06500 (10)	0.0512 (4)	
O5	0.63860 (17)	0.06593 (14)	0.23141 (16)	0.0828 (7)	
O6	0.5331 (2)	−0.04262 (17)	0.2091 (2)	0.1186 (10)	
O7	0.70321 (18)	−0.05719 (14)	0.21816 (15)	0.0844 (7)	
N1	0.29156 (15)	0.04450 (12)	0.26479 (12)	0.0397 (4)	
N2	0.46270 (15)	0.17344 (12)	0.24593 (12)	0.0427 (5)	
H2A	0.4009	0.1712	0.2025	0.051*	
H2B	0.5096	0.1336	0.2365	0.051*	
N3	0.03405 (15)	0.23032 (12)	0.20425 (13)	0.0434 (5)	
N4	−0.11498 (16)	0.07668 (13)	0.23973 (13)	0.0502 (5)	
H4A	−0.1721	0.0423	0.2152	0.060*	
H4B	−0.0579	0.0608	0.2202	0.060*	
N5	0.18116 (19)	0.15588 (14)	0.36497 (15)	0.0561 (6)	
N6	0.62424 (19)	−0.01249 (16)	0.22004 (15)	0.0592 (6)	
C1	0.18606 (19)	−0.06203 (13)	0.16659 (14)	0.0382 (5)	
C2	0.08048 (18)	−0.02498 (13)	0.15115 (14)	0.0357 (5)	
C3	−0.00785 (19)	−0.06361 (14)	0.09016 (14)	0.0401 (5)	
C4	0.0100 (2)	−0.13289 (15)	0.04369 (16)	0.0507 (6)	
H4	−0.0482	−0.1558	0.0015	0.061*	
C5	0.1139 (2)	−0.16862 (16)	0.05934 (18)	0.0561 (7)	
H5	0.1252	−0.2158	0.0280	0.067*	
C6	0.2000 (2)	−0.13502 (15)	0.12040 (17)	0.0501 (6)	
H6	0.2689	−0.1608	0.1316	0.060*	
C7	0.28236 (19)	−0.02800 (14)	0.22705 (15)	0.0411 (5)	
H7	0.3447	−0.0626	0.2401	0.049*	
C8	0.3959 (2)	0.06192 (16)	0.32864 (16)	0.0492 (6)	
H8A	0.4517	0.0230	0.3198	0.059*	
H8B	0.3869	0.0507	0.3846	0.059*	
C9	0.4351 (2)	0.15274 (16)	0.32568 (15)	0.0470 (6)	
H9A	0.3780	0.1916	0.3323	0.056*	
H9B	0.4997	0.1619	0.3731	0.056*	
C10	0.5139 (2)	0.26019 (17)	0.24785 (18)	0.0597 (7)	
H10A	0.4657	0.3026	0.2622	0.072*	
H10B	0.5205	0.2737	0.1916	0.072*	
C11	0.6256 (2)	0.26614 (19)	0.31053 (19)	0.0639 (8)	
H11A	0.6178	0.2663	0.3672	0.096*	
H11B	0.6612	0.3181	0.3009	0.096*	
H11C	0.6694	0.2178	0.3035	0.096*	
C12	−0.2001 (2)	−0.05535 (18)	0.01591 (16)	0.0537 (7)	
H12A	−0.2182	−0.1140	0.0266	0.064*	
H12B	−0.1828	−0.0535	−0.0382	0.064*	
C13	−0.2952 (2)	0.0027 (2)	0.01422 (19)	0.0710 (8)	
H13A	−0.3164	−0.0041	0.0658	0.107*	
H13B	−0.3562	−0.0117	−0.0334	0.107*	
H13C	−0.2739	0.0611	0.0091	0.107*	
C14	0.11326 (18)	0.30877 (14)	0.10555 (14)	0.0389 (5)	
C15	0.20996 (17)	0.26120 (13)	0.11291 (13)	0.0355 (5)	
C16	0.27758 (19)	0.28547 (15)	0.06010 (14)	0.0410 (5)	
C17	0.2535 (2)	0.35687 (16)	0.00929 (16)	0.0551 (7)	
H17	0.3005	0.3732	−0.0228	0.066*	
C18	0.1593 (2)	0.40514 (18)	0.00539 (17)	0.0599 (7)	
H18	0.1442	0.4542	−0.0282	0.072*	
C19	0.0899 (2)	0.38012 (16)	0.05081 (16)	0.0514 (6)	
H19	0.0252	0.4108	0.0458	0.062*	
C20	0.03422 (19)	0.29130 (15)	0.15277 (15)	0.0433 (6)	
H20	−0.0242	0.3297	0.1444	0.052*	
C21	−0.0571 (2)	0.23231 (17)	0.2451 (2)	0.0602 (7)	
H21A	−0.0905	0.2888	0.2372	0.072*	
H21B	−0.0267	0.2234	0.3057	0.072*	
C22	−0.1456 (2)	0.16612 (18)	0.2113 (2)	0.0644 (8)	
H22A	−0.2106	0.1821	0.2286	0.077*	
H22B	−0.1654	0.1676	0.1498	0.077*	
C23	−0.0843 (3)	0.06135 (19)	0.33260 (18)	0.0655 (8)	
H23A	−0.1491	0.0676	0.3531	0.079*	
H23B	−0.0307	0.1039	0.3610	0.079*	
C24	−0.0368 (3)	−0.0266 (2)	0.3540 (2)	0.0901 (11)	
H24A	−0.0897	−0.0688	0.3259	0.135*	
H24B	−0.0190	−0.0352	0.4143	0.135*	
H24C	0.0288	−0.0322	0.3355	0.135*	
C25	0.4284 (2)	0.2466 (2)	0.00521 (18)	0.0654 (8)	
H25A	0.4644	0.3021	0.0151	0.078*	
H25B	0.3800	0.2458	−0.0522	0.078*	
C26	0.5126 (3)	0.1772 (2)	0.0158 (2)	0.0878 (11)	
H26A	0.5575	0.1763	0.0735	0.132*	
H26B	0.5582	0.1878	−0.0214	0.132*	
H26C	0.4762	0.1229	0.0020	0.132*	
C27	0.2027 (2)	0.14706 (16)	0.43733 (18)	0.0491 (6)	

### Atomic displacement parameters (Å^2^)

**Table:**

	*U*^11^	*U*^22^	*U*^33^	*U*^12^	*U*^13^	*U*^23^
Zn1	0.03578 (15)	0.03238 (15)	0.04647 (17)	−0.00390 (11)	0.01557 (12)	−0.00297 (11)
S1	0.0852 (6)	0.1015 (7)	0.0522 (4)	−0.0016 (5)	0.0221 (4)	0.0014 (4)
O1	0.0345 (8)	0.0380 (9)	0.0611 (10)	−0.0048 (7)	0.0202 (8)	−0.0166 (7)
O2	0.0383 (9)	0.0521 (10)	0.0565 (10)	−0.0086 (8)	0.0135 (8)	−0.0118 (8)
O3	0.0348 (8)	0.0423 (9)	0.0514 (9)	0.0036 (7)	0.0148 (7)	0.0109 (7)
O4	0.0447 (10)	0.0605 (11)	0.0526 (10)	0.0014 (8)	0.0205 (8)	0.0112 (8)
O5	0.0583 (12)	0.0506 (13)	0.144 (2)	−0.0014 (10)	0.0361 (13)	−0.0036 (12)
O6	0.0652 (15)	0.1000 (19)	0.208 (3)	−0.0290 (14)	0.0664 (18)	−0.0322 (19)
O7	0.0703 (14)	0.0690 (14)	0.125 (2)	0.0200 (12)	0.0454 (14)	0.0028 (13)
N1	0.0351 (10)	0.0365 (11)	0.0471 (11)	−0.0016 (8)	0.0110 (9)	0.0076 (9)
N2	0.0353 (10)	0.0424 (11)	0.0471 (11)	−0.0054 (9)	0.0057 (9)	0.0008 (9)
N3	0.0396 (11)	0.0345 (11)	0.0600 (13)	−0.0019 (8)	0.0206 (9)	−0.0086 (9)
N4	0.0392 (11)	0.0513 (13)	0.0690 (14)	−0.0087 (9)	0.0301 (10)	−0.0145 (11)
N5	0.0614 (15)	0.0613 (15)	0.0481 (13)	−0.0093 (11)	0.0195 (11)	−0.0086 (11)
N6	0.0469 (13)	0.0566 (15)	0.0828 (17)	−0.0006 (11)	0.0327 (12)	0.0022 (12)
C1	0.0440 (13)	0.0289 (11)	0.0467 (13)	−0.0014 (10)	0.0209 (11)	0.0035 (10)
C2	0.0427 (12)	0.0284 (11)	0.0430 (12)	−0.0046 (9)	0.0237 (10)	−0.0008 (9)
C3	0.0451 (13)	0.0361 (12)	0.0439 (13)	−0.0058 (10)	0.0202 (11)	−0.0015 (10)
C4	0.0641 (17)	0.0418 (14)	0.0480 (14)	−0.0111 (12)	0.0183 (13)	−0.0100 (11)
C5	0.076 (2)	0.0350 (13)	0.0653 (17)	0.0021 (13)	0.0325 (15)	−0.0103 (12)
C6	0.0604 (16)	0.0337 (13)	0.0645 (17)	0.0086 (12)	0.0312 (14)	0.0036 (12)
C7	0.0399 (13)	0.0341 (13)	0.0534 (14)	0.0058 (10)	0.0199 (11)	0.0139 (11)
C8	0.0429 (14)	0.0505 (15)	0.0510 (15)	−0.0021 (11)	0.0072 (12)	0.0097 (12)
C9	0.0403 (13)	0.0503 (15)	0.0463 (14)	−0.0073 (11)	0.0050 (11)	0.0006 (11)
C10	0.0610 (17)	0.0486 (16)	0.0663 (18)	−0.0148 (13)	0.0121 (14)	0.0054 (13)
C11	0.0486 (16)	0.0622 (18)	0.081 (2)	−0.0182 (13)	0.0183 (15)	−0.0178 (15)
C12	0.0517 (15)	0.0602 (17)	0.0445 (14)	−0.0156 (13)	0.0056 (12)	0.0024 (12)
C13	0.0466 (16)	0.096 (2)	0.069 (2)	−0.0077 (16)	0.0136 (14)	0.0072 (17)
C14	0.0402 (13)	0.0331 (12)	0.0389 (12)	−0.0022 (10)	0.0033 (10)	−0.0076 (9)
C15	0.0357 (12)	0.0312 (12)	0.0353 (12)	−0.0056 (9)	0.0026 (9)	−0.0024 (9)
C16	0.0407 (13)	0.0423 (13)	0.0369 (12)	−0.0063 (10)	0.0053 (10)	−0.0010 (10)
C17	0.0661 (18)	0.0554 (16)	0.0433 (14)	−0.0082 (14)	0.0139 (13)	0.0093 (12)
C18	0.0716 (19)	0.0499 (16)	0.0526 (16)	0.0057 (14)	0.0074 (14)	0.0137 (13)
C19	0.0548 (16)	0.0429 (14)	0.0494 (15)	0.0095 (12)	0.0021 (13)	−0.0005 (11)
C20	0.0362 (12)	0.0352 (13)	0.0550 (14)	0.0027 (10)	0.0066 (11)	−0.0122 (11)
C21	0.0563 (16)	0.0442 (15)	0.093 (2)	0.0027 (12)	0.0424 (16)	−0.0065 (14)
C22	0.0434 (15)	0.0602 (18)	0.100 (2)	0.0053 (13)	0.0371 (16)	0.0020 (16)
C23	0.0709 (19)	0.0653 (19)	0.071 (2)	−0.0144 (15)	0.0375 (16)	−0.0140 (15)
C24	0.099 (3)	0.082 (2)	0.097 (3)	−0.001 (2)	0.041 (2)	0.019 (2)
C25	0.0595 (18)	0.083 (2)	0.0630 (18)	−0.0080 (16)	0.0326 (15)	0.0040 (15)
C26	0.080 (2)	0.084 (2)	0.121 (3)	0.002 (2)	0.064 (2)	0.004 (2)
C27	0.0440 (14)	0.0466 (15)	0.0622 (17)	−0.0063 (11)	0.0240 (13)	−0.0089 (12)

### Geometric parameters (Å, °)

**Table:**

Zn1—O1	1.9641 (15)	C9—H9A	0.9700
Zn1—O3	1.9890 (15)	C9—H9B	0.9700
Zn1—N5	2.030 (2)	C10—C11	1.502 (4)
Zn1—N3	2.1305 (19)	C10—H10A	0.9700
Zn1—N1	2.1351 (19)	C10—H10B	0.9700
S1—C27	1.622 (3)	C11—H11A	0.9600
O1—C2	1.316 (2)	C11—H11B	0.9600
O2—C3	1.365 (3)	C11—H11C	0.9600
O2—C12	1.438 (3)	C12—C13	1.497 (4)
O3—C15	1.310 (2)	C12—H12A	0.9700
O4—C16	1.370 (3)	C12—H12B	0.9700
O4—C25	1.432 (3)	C13—H13A	0.9600
O5—N6	1.243 (3)	C13—H13B	0.9600
O6—N6	1.209 (3)	C13—H13C	0.9600
O7—N6	1.223 (3)	C14—C15	1.405 (3)
N1—C7	1.278 (3)	C14—C19	1.408 (3)
N1—C8	1.464 (3)	C14—C20	1.446 (3)
N2—C9	1.477 (3)	C15—C16	1.423 (3)
N2—C10	1.496 (3)	C16—C17	1.373 (3)
N2—H2A	0.9000	C17—C18	1.393 (4)
N2—H2B	0.9000	C17—H17	0.9300
N3—C20	1.271 (3)	C18—C19	1.353 (4)
N3—C21	1.481 (3)	C18—H18	0.9300
N4—C23	1.482 (3)	C19—H19	0.9300
N4—C22	1.488 (3)	C20—H20	0.9300
N4—H4A	0.9000	C21—C22	1.510 (4)
N4—H4B	0.9000	C21—H21A	0.9700
N5—C27	1.149 (3)	C21—H21B	0.9700
C1—C6	1.404 (3)	C22—H22A	0.9700
C1—C2	1.410 (3)	C22—H22B	0.9700
C1—C7	1.441 (3)	C23—C24	1.500 (4)
C2—C3	1.411 (3)	C23—H23A	0.9700
C3—C4	1.375 (3)	C23—H23B	0.9700
C4—C5	1.383 (4)	C24—H24A	0.9600
C4—H4	0.9300	C24—H24B	0.9600
C5—C6	1.363 (4)	C24—H24C	0.9600
C5—H5	0.9300	C25—C26	1.493 (4)
C6—H6	0.9300	C25—H25A	0.9700
C7—H7	0.9300	C25—H25B	0.9700
C8—C9	1.505 (3)	C26—H26A	0.9600
C8—H8A	0.9700	C26—H26B	0.9600
C8—H8B	0.9700	C26—H26C	0.9600
			
O1—Zn1—O3	118.31 (7)	C10—C11—H11B	109.5
O1—Zn1—N5	113.14 (8)	H11A—C11—H11B	109.5
O3—Zn1—N5	128.40 (8)	C10—C11—H11C	109.5
O1—Zn1—N3	91.66 (7)	H11A—C11—H11C	109.5
O3—Zn1—N3	89.74 (7)	H11B—C11—H11C	109.5
N5—Zn1—N3	92.49 (9)	O2—C12—C13	107.4 (2)
O1—Zn1—N1	87.31 (7)	O2—C12—H12A	110.2
O3—Zn1—N1	87.34 (7)	C13—C12—H12A	110.2
N5—Zn1—N1	91.49 (8)	O2—C12—H12B	110.2
N3—Zn1—N1	175.98 (7)	C13—C12—H12B	110.2
C2—O1—Zn1	127.92 (13)	H12A—C12—H12B	108.5
C3—O2—C12	117.82 (19)	C12—C13—H13A	109.5
C15—O3—Zn1	129.83 (14)	C12—C13—H13B	109.5
C16—O4—C25	117.45 (19)	H13A—C13—H13B	109.5
C7—N1—C8	117.0 (2)	C12—C13—H13C	109.5
C7—N1—Zn1	122.93 (16)	H13A—C13—H13C	109.5
C8—N1—Zn1	120.08 (15)	H13B—C13—H13C	109.5
C9—N2—C10	112.61 (19)	C15—C14—C19	119.8 (2)
C9—N2—H2A	109.1	C15—C14—C20	124.4 (2)
C10—N2—H2A	109.1	C19—C14—C20	115.7 (2)
C9—N2—H2B	109.1	O3—C15—C14	124.2 (2)
C10—N2—H2B	109.1	O3—C15—C16	118.4 (2)
H2A—N2—H2B	107.8	C14—C15—C16	117.3 (2)
C20—N3—C21	115.3 (2)	O4—C16—C17	124.3 (2)
C20—N3—Zn1	123.00 (16)	O4—C16—C15	114.78 (19)
C21—N3—Zn1	121.50 (17)	C17—C16—C15	120.9 (2)
C23—N4—C22	116.4 (2)	C16—C17—C18	120.7 (3)
C23—N4—H4A	108.2	C16—C17—H17	119.7
C22—N4—H4A	108.2	C18—C17—H17	119.7
C23—N4—H4B	108.2	C19—C18—C17	119.5 (2)
C22—N4—H4B	108.2	C19—C18—H18	120.3
H4A—N4—H4B	107.4	C17—C18—H18	120.3
C27—N5—Zn1	163.6 (2)	C18—C19—C14	121.6 (2)
O6—N6—O7	121.5 (3)	C18—C19—H19	119.2
O6—N6—O5	119.9 (2)	C14—C19—H19	119.2
O7—N6—O5	118.6 (2)	N3—C20—C14	128.5 (2)
C6—C1—C2	119.2 (2)	N3—C20—H20	115.7
C6—C1—C7	117.5 (2)	C14—C20—H20	115.7
C2—C1—C7	123.3 (2)	N3—C21—C22	114.1 (2)
O1—C2—C1	123.1 (2)	N3—C21—H21A	108.7
O1—C2—C3	118.5 (2)	C22—C21—H21A	108.7
C1—C2—C3	118.4 (2)	N3—C21—H21B	108.7
O2—C3—C4	125.2 (2)	C22—C21—H21B	108.7
O2—C3—C2	114.18 (19)	H21A—C21—H21B	107.6
C4—C3—C2	120.6 (2)	N4—C22—C21	115.1 (2)
C3—C4—C5	120.3 (2)	N4—C22—H22A	108.5
C3—C4—H4	119.8	C21—C22—H22A	108.5
C5—C4—H4	119.8	N4—C22—H22B	108.5
C6—C5—C4	120.3 (2)	C21—C22—H22B	108.5
C6—C5—H5	119.8	H22A—C22—H22B	107.5
C4—C5—H5	119.8	N4—C23—C24	111.2 (2)
C5—C6—C1	121.0 (2)	N4—C23—H23A	109.4
C5—C6—H6	119.5	C24—C23—H23A	109.4
C1—C6—H6	119.5	N4—C23—H23B	109.4
N1—C7—C1	127.2 (2)	C24—C23—H23B	109.4
N1—C7—H7	116.4	H23A—C23—H23B	108.0
C1—C7—H7	116.4	C23—C24—H24A	109.5
N1—C8—C9	112.99 (19)	C23—C24—H24B	109.5
N1—C8—H8A	109.0	H24A—C24—H24B	109.5
C9—C8—H8A	109.0	C23—C24—H24C	109.5
N1—C8—H8B	109.0	H24A—C24—H24C	109.5
C9—C8—H8B	109.0	H24B—C24—H24C	109.5
H8A—C8—H8B	107.8	O4—C25—C26	108.6 (2)
N2—C9—C8	113.2 (2)	O4—C25—H25A	110.0
N2—C9—H9A	108.9	C26—C25—H25A	110.0
C8—C9—H9A	108.9	O4—C25—H25B	110.0
N2—C9—H9B	108.9	C26—C25—H25B	110.0
C8—C9—H9B	108.9	H25A—C25—H25B	108.4
H9A—C9—H9B	107.8	C25—C26—H26A	109.5
N2—C10—C11	112.9 (2)	C25—C26—H26B	109.5
N2—C10—H10A	109.0	H26A—C26—H26B	109.5
C11—C10—H10A	109.0	C25—C26—H26C	109.5
N2—C10—H10B	109.0	H26A—C26—H26C	109.5
C11—C10—H10B	109.0	H26B—C26—H26C	109.5
H10A—C10—H10B	107.8	N5—C27—S1	179.6 (3)
C10—C11—H11A	109.5		

### Hydrogen-bond geometry (Å, °)

**Table:**

*D*—H···*A*	*D*—H	H···*A*	*D*···*A*	*D*—H···*A*
N2—H2B···O5	0.90	1.96	2.843 (3)	166
N2—H2A···O3	0.90	1.98	2.772 (2)	146
N2—H2A···O4	0.90	2.37	3.022 (3)	129
N4—H4A···O7^i^	0.90	2.22	3.050 (3)	153
N4—H4A···O5^i^	0.90	2.50	3.080 (3)	122
N4—H4B···O1	0.90	1.71	2.606 (2)	171
N4—H4B···O2	0.90	2.55	3.051 (3)	116

Symmetry codes: (i) *x*−1, *y*, *z*.

## References

[bb1] Berg, J. M. & Shi, Y. (1996). *Science*, **271**, 1081–1985. 10.1126/science.271.5252.1081 8599083

[bb2] Bruker (1998). *SMART* and *SAINT* Bruker AXS Inc., Madison, Wisconsin, USA.

[bb3] Cai, J.-H., Huang, Y.-H. & Jiang, Y.-M. (2006). *Acta Cryst.* E**62**, m2432–m2434.

[bb4] Chohan, Z. H. & Kausar, S. (1993). *Chem. Pharm. Bull.* **41**, 951–953. 10.1248/cpb.41.951 8339341

[bb5] Correia, I., Dornyei, A., Avecilla, F., Kiss, T. & Pessoa, J. C. (2006). *Eur. J. Inorg. Chem.* pp. 656–662.

[bb6] Eltayeb, N. E., Teoh, S. G., Chantrapromma, S., Fun, H.-K. & Adnan, R. (2008). *Acta Cryst.* E**64**, m912–m913. 10.1107/S1600536808017340 PMC2961853 21202773

[bb7] Ghosh, R., Rahaman, S. H., Lin, C.-N., Lu, T.-H. & Ghosh, B. K. (2006). *Polyhedron*, **25**, 3104–3112.

[bb8] Odoko, M., Tsuchida, N. & Okabe, N. (2006). *Acta Cryst.* E**62**, m710–m711.

[bb9] Osowole, A. A., Kolawole, G. A. & Fagade, O. E. (2008). *J. Coord. Chem.* **61**, 1046–1055.

[bb10] Samanta, B., Chakraborty, J., Shit, S., Batten, S. R., Jensen, P., Masuda, J. D. & Mitra, S. (2007). *Inorg. Chim. Acta*, **360**, 2471–2484.

[bb11] Sheldrick, G. M. (1996). *SADABS* University of Göttingen, Germany.

[bb12] Sheldrick, G. M. (2008). *Acta Cryst.* A**64**, 112–122. 10.1107/S0108767307043930 18156677

[bb13] Tarafder, M. T. H., Chew, K.-B., Crouse, K. A., Ali, A. M., Yamin, B. M. & Fun, H.-K. (2002). *Polyhedron*, **21**, 2683–2690.

[bb14] Tatar, L., Atakol, O. & Ülkü, D. (2002). *Acta Cryst.* E**58**, m83–m85.

[bb15] Zhang, S.-H., Feng, X.-Z., Li, G.-Z., Jing, L.-X. & Liu, Z. (2007). *Acta Cryst.* E**63**, m396–m398.

